# Role of Common Genetic Variants for Drug-Resistance to Specific Anti-Seizure Medications

**DOI:** 10.3389/fphar.2021.688386

**Published:** 2021-06-09

**Authors:** Stefan Wolking, Ciarán Campbell, Caragh Stapleton, Mark McCormack, Norman Delanty, Chantal Depondt, Michael R. Johnson, Bobby P. C. Koeleman, Roland Krause, Wolfram S. Kunz, Anthony G. Marson, Josemir W. Sander, Graeme J. Sills, Pasquale Striano, Federico Zara, Sanjay M. Sisodiya, Gianpiero L. Cavalleri, Holger Lerche

**Affiliations:** ^1^ Neurology and Epileptology, Hertie Institute for Clinical Brain Research, University of Tübingen, Tübingen, Germany; ^2^ Department of Epileptology and Neurology, University of Aachen, Aachen, Germany; ^3^ Department of Molecular and Cellular Therapeutics, Royal College of Surgeons in Ireland, Dublin, Ireland; ^4^ FutureNeuro Research Centre, Science Foundation Ireland, Dublin, Ireland; ^5^ Division of Neurology, Beaumont Hospital, Dublin, Ireland; ^6^ Department of Neurology, Hôpital Erasme, Université Libre de Bruxelles, Brussels, Belgium; ^7^ Division of Brain Sciences, Imperial College Faculty of Medicine, London, United Kingdom; ^8^ Department of Genetics, University Medical Center Utrecht, Utrecht, Netherlands; ^9^ Luxembourg Centre for Systems Biomedicine, University of Luxembourg, Esch-sur-Alzette, Luxembourg; ^10^ Institute of Experimental Epileptology and Cognition Research and Department of Epileptology, University of Bonn, Bonn, Germany; ^11^ Department of Molecular and Clinical Pharmacology, Institute of Translational Medicine, University of Liverpool, Liverpool, United Kingdom; ^12^ The Walton Centre NHS Foundation Trust, Liverpool, United Kingdom; ^13^ Liverpool Health Partners, Liverpool, United Kingdom; ^14^ Department of Clinical and Experimental Epilepsy, UCL Queen Square Institute of Neurology, London, United Kingdom; ^15^ Chalfont Centre for Epilepsy, Chalfont-St-Peter, United Kingdom; ^16^ Stichting Epilepsie Instellingen Nederland (SEIN), Heemstede, Netherlands; ^17^ School of Life Sciences, University of Glasgow, Glasgow, United Kingdom; ^18^ IRCCS "G. Gaslini" Institute, Genova, Italy; ^19^ Department of Neurosciences, University of Genoa, Genova, Italy

**Keywords:** drug-resistant epilepsies, polygenic risk score (PRS), GWAS, anti-seizure medication (ASM), single nucelotide polymorphisms

## Abstract

**Objective:** Resistance to anti-seizure medications (ASMs) presents a significant hurdle in the treatment of people with epilepsy. Genetic markers for resistance to individual ASMs could support clinicians to make better-informed choices for their patients. In this study, we aimed to elucidate whether the response to individual ASMs was associated with common genetic variation.

**Methods:** A cohort of 3,649 individuals of European descent with epilepsy was deeply phenotyped and underwent single nucleotide polymorphism (SNP)-genotyping. We conducted genome-wide association analyses (GWASs) on responders to specific ASMs or groups of functionally related ASMs, using non-responders as controls. We performed a polygenic risk score (PRS) analyses based on risk variants for epilepsy and neuropsychiatric disorders and ASM resistance itself to delineate the polygenic burden of ASM-specific drug resistance.

**Results:** We identified several potential regions of interest but did not detect genome-wide significant loci for ASM-specific response. We did not find polygenic risk for epilepsy, neuropsychiatric disorders, and drug-resistance associated with drug response to specific ASMs or mechanistically related groups of ASMs.

**Significance:** This study could not ascertain the predictive value of common genetic variants for ASM responder status. The identified suggestive loci will need replication in future studies of a larger scale.

## Introduction

About one-third of people with epilepsy have seizures that are refractory to anti-seizure medications (ASMs). The International League against Epilepsy (ILAE) defines drug resistance as ongoing seizures despite treatment with at least two well-tolerated and appropriate ASMs (Kwan et al., 2010). With each additional drug trial, the odds to achieve seizure freedom decrease (Brodie et al., 2012). The introduction of new ASMs with alternate mechanisms of action has not significantly changed this situation (Chen et al., 2018). For certain epilepsy syndromes, some ASMs have proven to be more beneficial than others: valproic acid (VPA) and ethosuximide are superior to lamotrigine (LTG) in childhood absence epilepsy (Glauser et al., 2013), VPA is superior to topiramate (TPM) and LTG in genetic generalized epilepsy (GGE) (Marson et al., 2007a; Silvennoinen et al., 2019), and carbamazepine (CBZ) and LTG are superior to TPM and gabapentin (GBP) in focal epilepsy (Marson et al., 2007b). Yet, studies with head-to-head comparisons are sparse (Beyenburg et al., 2010; Androsova et al., 2017). Thus, in clinical practice, ASMs are prescribed based on age, gender, co-morbidities, electroclinical syndrome, seizure type, potential drug interactions, or adverse drug reactions.

Pharmacogenomics, i.e. the influence of genetic variants on drug response or adverse effects, bear the potential to support the choice of the most suitable ASM (Löscher et al., 2009). Other medical fields have seen the integration of pharmacogenomics in clinical routine (Daly, 2017). For epilepsies, reproducible pharmacogenomic findings are limited to cutaneous adverse reactions caused by aromatic ASMs (Chung et al., 2004; McCormack et al., 2011; McCormack et al., 2018). The utility of these findings in individuals’ care remains a matter of debate (Chen et al., 2014). The endeavor to identify common genetic variants associated with drug response is still elusive, also due to small sample sizes (Heavin et al., 2019; Wolking et al., 2020a). There is some evidence that enrichment of ultra-rare variants in genes associated with pharmacodynamics and pharmacokinetics can modify ASM response, but further replication of these results is needed (Wolking et al., 2020a).

We assessed common variants' role and common variant burden for drug response to common ASMs using genome-wide association studies (GWAS) and polygenic risk score (PRS) analyses in a cohort of 3,649 individuals.

## Methods

### Ethics Statement

All study participants provided written, informed consent for genetic analyses. Local institutional review boards reviewed and approved study protocols at each contributing site.

### Study Design

This cohort was derived from the EpiPGX Consortium established in 2012 to identify genetic biomarkers of epilepsy treatment response and adverse drug reactions. EpiPGX is a European-wide epilepsy research partnership under the European Commission Seventh Framework Protocol (FP7). This case-control study was based on the retrospective evaluation of individual data. Relevant data were extracted from case charts by trained personnel and collected in a standard electronic case report form (eCRF) used at all consortium sites. Our cohorts consisted exclusively of individuals of non-Finnish European ancestry with an established diagnosis of either focal or genetic generalized epilepsy according to current ILAE diagnostic criteria (Scheffer et al., 2017). We tested whether common genetic variants were significantly associated with drug response to one ASM or groups of mechanistically related ASMs (sodium channel-active and calcium channel-active ASMs). We also tested whether the response profile was associated with an increased burden of polygenic variants for risk of epilepsy syndromes, other neuropsychiatric disorders, or whether a burden of risk variants for drug response itself could predict the outcome.

ASMs were selected based on their usage in the EpiPGX cohort. ASM-specific analysis was performed for levetiracetam (LEV), lamotrigine (LTG), valproic acid (VPA) for focal epilepsies and all epilepsies. For focal epilepsies only, we performed additional ASM-specific GWAS for phenytoin (PHT), oxcarbazepine (OXC), and carbamazepine (CBZ). ASM groups comprised T-type calcium channel-active ASMs (valproic acid, ethosuximide, and zonisamide [ZNS]) for focal and all epilepsies; and sodium channel-active ASMs (LTG, lacosamide [LCM], ZNS, PHT, CBZ, OXC, and eslicarbazepine [ESL]) for focal epilepsies only. The breakdown of the sample size per analysis is depicted in Table 1.

**TABLE 1:** Sample numbers, estimated power, and clinical details for GWAS cohorts.

ASM	Status	*n*	Study power	Female (%)	GGE (%)	AOO (mean, SD)	Ethnicity %		
							South Europe	Central Europe	British Isles
LEV	R	343	1.55	58.9	24.8	24.7 (±19.0)	10.2	32.7	57.1
	N	895		56.6	24.2	18.2 (±14.8)	9.1	43.4	47.6
Na-C-ASMs	R	910	1.37	50.7	0	30.9 (±19.7)	14.6	17.5	67.9
	N	1,286		54.2	0	21.5 (±16.5)	6.5	31.5	62.0
LTG	R	471	1.49	58.0	29.1	26.3 (±19.0)	7.5	36.5	56.0
	N	929		61.9	26.8	19.1 (±15.2)	6.8	40.5	52.7
CBZ	R	424	1.57	47.6	0	30.4 (±19.6)	21.7	12.0	66.3
	N	591		55.8	0	20.7 (±16.6)	13.7	27.2	59.1
OXC	R	98	2.08	55.1	0	29.6 (±19.6)	21.4	15.3	63.3
	N	296		50.7	0	18.1 (±14.1)	10.1	48.6	41.2
PHT	R	71	2.30	47.9	0	28.2 (±20.3)	14.1	18.3	67.6
	N	218		54.1	0	18.0 (±14.6)	15.6	20.2	64.2
Ca-C-ASMs	R	690	1.45	59.3	69.0	16.6 (±14.2)	14.6	54.1	31.3
	N	848		51.7	20.6	18.9 (±15.3)	9.4	39.6	50.9
VPA	R	612	1.49	56.9	67.0	17.6 (±14.5)	15.4	53.9	30.7
	N	690		51.3	23.5	20.0 (±16.0)	8.4	44.5	47.1
All samples	—	3,649		55.0	24.3	22.8 (±17.7)	8.9	34.8	56.4

Depiction of sample size per ASM and responder status, study power, gender distribution, mean age at seizure onset, and distribution of ethnicity. Study power shows relative risk for 80% study power, given an allele frequency of ≥20%, an α level of 5 × 10–8 and a prevalence of drug-resistance of 30%. AOO = age of onset of first seizure, ASM = anti-seizure medication, Ca-C-ASMs = T-type calcium channel-active anti-seizure medications, CBZ = carbamazepine, GGE = genetic generalized epilepsy, LEV = levetiracetam, LTG = lamotrigine, n = number, N = non-responders, Na-C-ASMs = sodium channel-active anti-seizure medications, OXC = oxcarbazepine, PHT = phenytoin, R = responders, SD = standard deviation, VPA = valproic acid. Ca-C-ASMs comprised VPA, zonisamide, and ethosuximide; Na-C-ASMs comprised LTG, lacosamide, zonisdamide, PHT, CBZ, OXC, and eslicarbazepine.

### Cohorts and Phenotype Definition

The individuals in this study were selected from more than 12.000 individuals that were documented in the EpiPGX eCRF. Thereof, 3,649 individuals fulfilled the inclusion criteria, 2,762 with focal epilepsy, and 887 with generalized genetic epilepsy. The latter group has been part of a previous study (Wolking et al., 2020a). A more detailed cohort description is provided in Table 1.

Individuals were classified as responders or non-responders. The response was defined as seizure freedom under ongoing treatment for at least one year and before initiation of any other treatment; non-response as recurring seizures at ≥ 50% of pretreatment seizure frequency given adequate dosage of the trial drug. Individuals with recurrent non-compliance for ASM intake were excluded. The response or non-response groups' assignment was based on the evaluation of one or more epilepsy specialists at the source center. To harmonize phenotyping procedures a phenotyping manual was created at the start of the EpiPGX project. At the beginning and on a yearly basis throughout the recruitment phase phenotyping workshops were held. To assess cross-center consistency of data interpretation, a cross-center phenotyping validation test was performed at the outset of the EpiPGX project, using anonymized medical records. An overall inter-rater agreement of 74.2% was reached. Stark disagreement, e.g ASM response vs. non-response, occurred in 5.1% of recorded ASM trials.

### Imputation and Genotyping Quality Controls

GWASs were conducted separately for each ASM-response cohort using imputed genotypes. Genotyping of a subset of samples was performed at deCODE Genetics on Illumina OmniExpress-12 v1.1 and -24 v1.1 single nucleotide polymorphism (SNP) arrays. The remainder of samples were genotyped locally on various Illumina beadchip SNP arrays. Detailed genotyping, imputation and quality control methods have been described previously (McCormack et al., 2018). Population structure was controlled via principal component analysis as reported previously (Wolking et al., 2020a) (Supplementary Figure S1). European ancestry was established by a principal component analysis comparison to 1,000 genomes data (Supplementary Figure S2).

### Genome-wide Association Analysis

GWAS power was calculated using PGA (Menashe et al., 2008). Association analysis was performed using SNPTEST in a frequentist model with the top 10 main components, sex, and epilepsy subtype (where appropriate) included as covariates. The statistical threshold for genome-wide significance was set at *p* < 5 × 10^–8^. Post-association QC removed SNPs with INFO scores lower than 0.95, missingness rates >0.10, Hardy-Weinberg deviations *p* < 5 × 10^–6^, and minor allele frequencies <5%.

### Study Power

We estimated that our most extensive analysis for sodium channel-active ASMs had 80% power to detect a genetic predictor of the relative risk of 1.37 with an allele frequency of ≥20%, based on an *α* level of 5 × 10^–8^ and given a prevalence of drug-resistance of 30%. The study power for the other analyses is shown in Table 1.

### Polygenic Risk Score Analysis for Epilepsy and Neuropsychiatric Disorders

GWAS summary statistics for epilepsy (focal, GGE, and all epilepsies) were downloaded from the ILAE study (Cross-Disorder Group of the Psychiatric Genomics Consortium, 2013) using the EpiGAD server. These statistics were remade with the overlapping samples between the larger ILAE cohort and our EpiPGX samples removed. GWAS results for a broad psychiatric disorder study (covering autism, attention deficit hyperactivity disorder, bipolar disorder, major depression and schizophrenia) were downloaded from the psychiatric genomics consortium (International League Against Epilepsy Consortium on Complex Epilepsies, 2018). PRS for each phenotype were calculated for all samples our study cohorts using PRSice (Euesden et al., 2015), using all SNPs from the base GWAS with *p*-values ≤0.5. The threshold of ≤0.5 was selected because for most complex traits the most predictive p-thresholds will typically be between 0.3 and 0.5, including epilepsy (Leu et al., 2019). The PRS were then regressed onto responder status using R 3.6, with the top six principle components, sex, and epilepsy subtype (where appropriate) included as covariates.

### Polygenic Risk Score Analysis for Drug Response

To test whether responsiveness to individuals ASMs or groups of ASMs had a distinct polygenic component, we split our cohorts into a discovery and replication cohort, depending on recruitment site (Test: 636 cases, 890 controls; discovery: 229 cases, 323 controls). A GWAS was run in the test cohort (following the protocol from above) and used a PRS analysis base in the discovery cohort (same methods as above).

### SNP-Heritability Testing

Linkage disequilibrium score-regression (Bulik-Sullivan et al., 2015) was used to calculate SNP-based heritability in the cohort of sodium-channel actives ASM treated study participants. We also used GCTA-GREML to estimate the heritability (Yang et al., 2011).

## Results

### Cohort Description

After per individual quality check, 3,649 individuals were included in the GWAS analyses. The breakdown of the GWAS cohorts is shown in Table 1. The proportion of individuals with GGE was 25%. For the GWAS for VPA response and in consequence for T-type calcium channel-active ASMs (including VPA, ESX, and ZNS), GGE was overrepresented in the responder group. The mean age of onset tended to be higher for responders than non-responders except for VPA and T-type calcium channel-active ASMs.

### Genome-wide Association Studies for Drug Response

**FIGURE 1:**
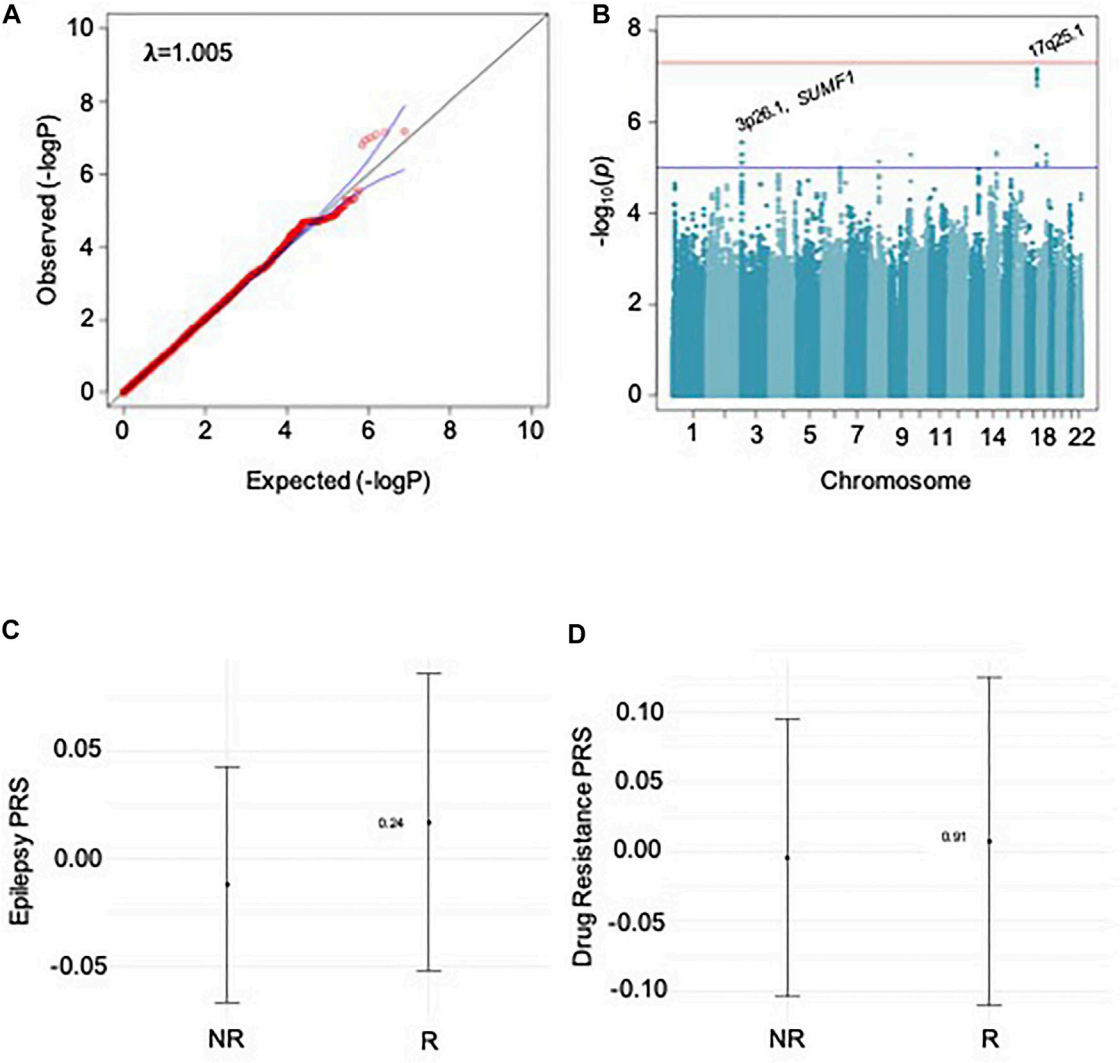
Results for sodium channel-active ASMs. A and B show results for GWAS, C and D for PRS analysis. A: QQ plot shows no relevant p-value inflation; lambda-value = 1.005. B: Manhattan plot of GWAS; locations of SNPs with p-value ≤ 5 × 10–6 are marked. C: Results for PRS-analysis PRS for epilepsy risk based on the ILAE 2018 metadata. D: Results for PRS-analysis for PRS for risk of ASM-specific drug response.

We performed GWAS for drug response for specific ASMs and groups of ASMs (as shown in Table 1) for focal epilepsy and all epilepsies. Results for GGE alone have been published previously (Wolking et al., 2020b). We found no evidence for a relevant GWAS *p*-value inflation (lambda-range between 0.99 and 1.06). We did not find any genome-wide markers that exceeded the significance threshold (5 × 10^–8^). We identified 30 loci suggestive for an association with ASM response (<5 × 10^–6^) as shown in Table 2. To exemplify the findings, QQ- and Manhattan plots for the largest GWAS of sodium channel-active ASMs are shown in Figures 1A,B; the results of the other GWAS are depicted in Supplementary Figures S3 to S12.

**TABLE 2:** Top genome-wide association study results (p < 5 × 10–6) for ASM responder status

SNP	Location (hg19)	p-value	Gene
Focal Epilepsies			
Levetiracetam			
rs10191428	2:62,725,407	2.37 × 10–6	TMEM17
rs6455984	6:1,65,419,809	2.98 × 10–6	—
rs10786411	10:100091761	4.01 × 10–6	—
Sodium channel-Active ASMs			
rs2600151	3:4148058	2.83 × 10–6	SUMF1
rs60350499	17:71111631	6.89 × 10–8	—
Lamotrigine			
rs7811069	7:32,003,223	1.75 × 10–6	PDE1C
rs1859577	7:68254624	4.80 × 10–7	—
rs2028234	8:4747736	6.90 × 10–7	CSMD1
Carbamazepine			
rs4078065	2:238110123	3.88 × 10–6	—
rs13150739	4:128045535	8.95 × 10–7	—
rs4243569	14:51536146	4.49 × 10–6	TRIM9
Oxcarbazepine			
rs6552076	4:68014557	4.71 × 10–6	—
rs1816237	5:33040812	1.00 × 10–6	—
rs2944715	8:69346689	3.10 × 10–6	C8orf34
rs34744859	18:65165115	4.44 × 10–6	—
Phenytoin			
rs12038219	1:167503917	6.07 × 10–8	—
rs28740860	3:3277529	8.72 × 10–7	—
rs188002	6:140473067	4.60 × 10–7	—
rs16945236	15:91664327	8.36 × 10–7	—
Calcium channel-Active ASMs			
rs11125398	2:52227824	2.77 × 10–6	—
rs73104283	2:231,130,300	3.64 × 10–6	SP140
rs7092992	10:20,922,643	3.56 × 10–6	—
Valproic acid			
rs2700204	3:112,841,569	4.52 × 10–6	—
rs1952670	9:128,654,392	9.11 × 10–7	PBX3
rs7092992	10:20,922,643	4.07 × 10–6	—
All Epilepsies			
Levetiracetam			
rs10191428	2:62,725,407	2.30 × 10–6	TMEM17
rs9390556	6:148,643,960	4.80 × 10–6	—
Lamotrigine			
rs12468936	2:106,116,654	2.60 × 10–6	—
rs7811069	7:32,003,223	8.44 × 10–7	PDE1C
rs7859863	9:104,337,744	4.52 × 10–6	GRIN3A
rs28776624	14:41,898,817	3.90 × 10–6	—
Calcium channel Active ASMs			
rs73104283	2:231,130,300	1.14 × 10–6	SP140
Valproic acid			
rs3936663	4:7,185,699	3.83 × 10–6	--

### SNP-Heritability Testing

We calculated SNP-based heritability [SNP-h^2^] as a measure of the proportion of variance in ASM response status, which could be attributed to common genetic variants for the largest cohort of samples treated with sodium-blocking ASMs. The result was not significant, SNP-h^2^ was estimated to be 0.3108, with a standard error of 0.2868 (Lower CI: −0.252, Upper CI: 0.873). Using GCTA-GREML to calculate h^2^, the result was not significant [h^2^ = 0.000002, standard error = 0.178,925, *p* = 0.5].

### Assessing the Polygenic Risk of Epilepsy and Neuropsychiatric Disorders for Drug Response

First, we tested whether the responder status to individual ASMs and the groups of sodium channel active-, and T-type calcium channel-active ASMs correlated with the genetic load for epilepsy (focal, generalized, and combined), Figure 1C. Second, we tested whether the responder status correlated with the genetic load for five neuropsychiatric disorders was associated with responder status. In both cases, we found no significant association of polygenic risk scores with any ASM drug-responder status.

Third, we assumed that the responder status itself harbored a polygenic component, which is largely distinct from the polygenic component for epilepsy risk. We split the ASM cohorts in half to calculate a GWAS for the first half. This discovery cohort was used to calculate PRS for individual ASM responder status in the second half. We also did not find a significant association for drug response PRS with responder status (Figure 1D).

## Discussion

We tested whether common genetic variation could predict drug response to various commonly used ASMs. We identified several loci of potential interest for ASM response but found no significant genome-wide association. Our analysis was underpowered to detect small effect size variants, but the results suggest that there are no large-effect size variants associated with drug response. We further tested whether the polygenic burden for epilepsy risk, risk for various neuropsychiatric disorders, or drug-resistance itself had a predictive value for the drug response phenotype. We could not show that polygenic risk scores were significantly associated with ASM response within the limits of study size. Other methods of PRS calculation also exist, such as LDpred (Vilhjálmsson et al., 2015), which may prove more successful at finding polygenic signals associated with drug response to ASMs and could be further explored in future studies.

This study was limited to the sample size of the sub-analyses. This study does not prove that drug response is without genetic influence. The results could imply that drug response is a far more complex trait with multiple influencing parameters beyond genomic factors alone. While PRS for epilepsy is a reliable predictor for the risk of epilepsy and epilepsy sub-phenotypes itself (Leu et al., 2019; Moreau et al., 2020), this approach was not beneficial to predict drug response within this study's limitations.

The results align with our previous studies that found no common genetic variants in association to VPA, LTG, and LEV response in genetic generalized epilepsy (Wolking et al., 2020b) or for the response to lacosamide in focal epilepsy (Heavin et al., 2019). One previous study suggests that rare genetic variants in genes related to drug targets and pharmacokinetics might be involved (Wolking et al., 2020b). Given that many individuals with epilepsy exhibit a broad pharmacoresistance, regardless of the drugs’ mechanism of action, other factors are probably at play (Löscher et al., 2020). Epigenetic mechanisms such as altered DNA methylation (Kobow et al., 2013), seizure-induced alterations of neural networks (Fang et al., 2011), or intrinsic factors mediating disease severity (Rogawski, 2013) should be further explored.

## Conclusion

No genome-wide significant variants could be identified in association with drug response to various widely used ASMs. We identified several suggestive risk loci. Future hypothesis-driven association studies should attempt to reproduce our findings.

## EpiPGX-CONSORTIUM

Andreja Avbersek, Costin Leu, Kristin Heggeli, Rita Demurtas, Joseph Willis, Douglas Speed, Narek Sargsyan, Krishna Chinthapalli, Mojgansadat Borghei, Antonietta Coppola, Antonio Gambardella, Stefan Wolking, Felicitas Becker, Sarah Rau, Christian Hengsbach, Yvonne G. Weber, Bianca Berghuis, Wolfram S. Kunz, Mark McCormack, Norman Delanty, Ellen Campbell, Lárus J. Gudmundsson, Andres Ingason, Kári Stefánsson, Reinhard Schneider, Rudi Balling, Pauls Auce, Ben Francis, Andrea Jorgensen, Andrew Morris, Sarah Langley, Prashant Srivastava, Martin Brodie, Marian Todaro, Slave Petrovski, Jane Hutton, Fritz Zimprich, Martin Krenn, Hiltrud Muhle, Karl Martin Klein, Rikke S Møller, Marina Nikanorova, Sarah Weckhuysen, Zvonka Rener-Primec, Gianpiero L. Cavalleri, John Craig, Chantal Depondt, Michael R. Johnson, Bobby P. C. Koeleman, Roland Krause, Holger Lerche, Anthony G. Marson, Terence J. O’Brien, Slave Petrovski, Samuel F. Berkovic, Josemir W. Sander, Graeme J. Sills, Hreinn Stefansson, Pasquale Striano, Federico Zara, and Sanjay M. Sisodiya

## Data Availability

The original contributions presented in the study are included in the article/Supplementary Material. The raw SNP datasets presented in this article are not readily available due to ethical and privacy restrictions. Further inquiries should be directed to the corresponding author.
