# Supplementary material for: Role of Common Genetic Variants for Drug-Resistance to Specific Anti-Seizure Medications
Source: Front Pharmacol. 2021 Jun 9;12:688386. doi: 10.3389/fphar.2021.688386 (PMC8220970; doi:10.3389/fphar.2021.688386)
Supplement: Supplementary file 1 [file DataSheet1.docx]

# Supplementary Material

## Recruitment Sites

Patients were recruited from the following tertiary epilepsy referral centers: University Hospital of Antwerp, Belgium; University Hospital Bonn, Bonn, Germany; Erasmus Hospital, Brussels, Belgium; Danish Epilepsy Centre, Filadelfia, Dianalund, Denmark; Beaumont Hospital and St. James’ Hospital, Royal College of Surgeons, Dublin, Ireland; Institute "G. Gaslini", University of Genova, Genoa, Italy; Epilepsy Unit, West Glasgow ACH-Yorkhill, UK; Gasthuisberg Hospital, Leuven, Belgium; The Walton Centre NHS Foundation Trust, Liverpool, UK; National Hospital for Neurology and Neurosurgery, London, UK; University Hospital of Marburg, Germany; University Hospital Tübingen, Tübingen, Germany; Department of Neurology, Vienna General Hospital, Vienna, Austria; Stichting Epilepsy Center, Heemstede, Netherlands.

### Figure S1: PCA of study subjects by country of origin


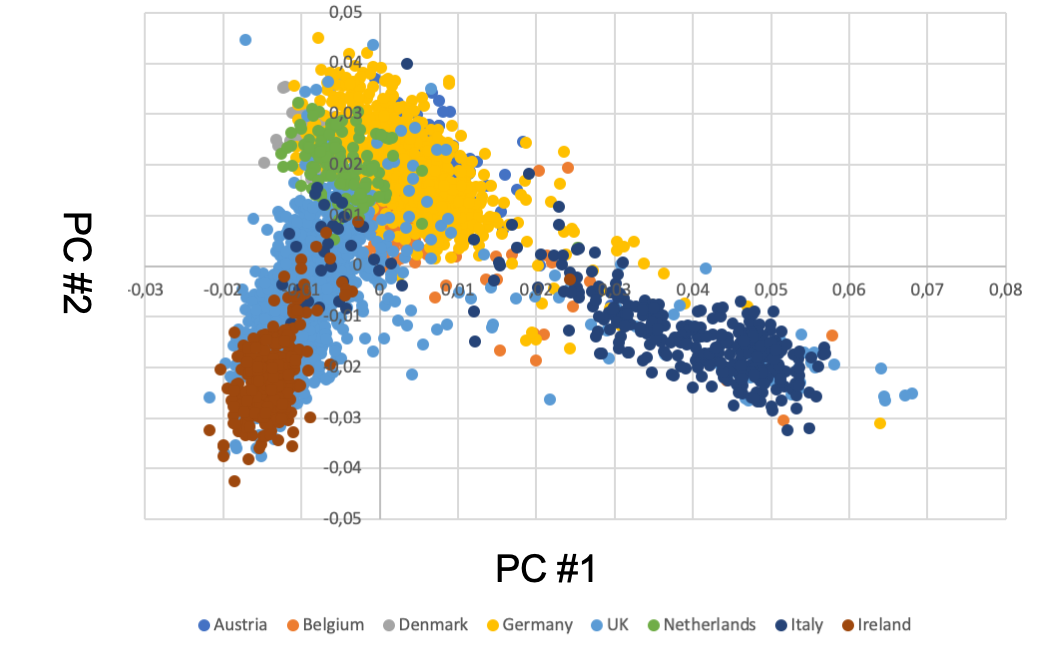


Figure S1: Scatter plot of first 2 principal components of all 3,649 study participants with respective country of origin. Plot shows no relevant outliers.

### Figure S2: PCA comparison of study subjects to the 1000 Genomes Project populations


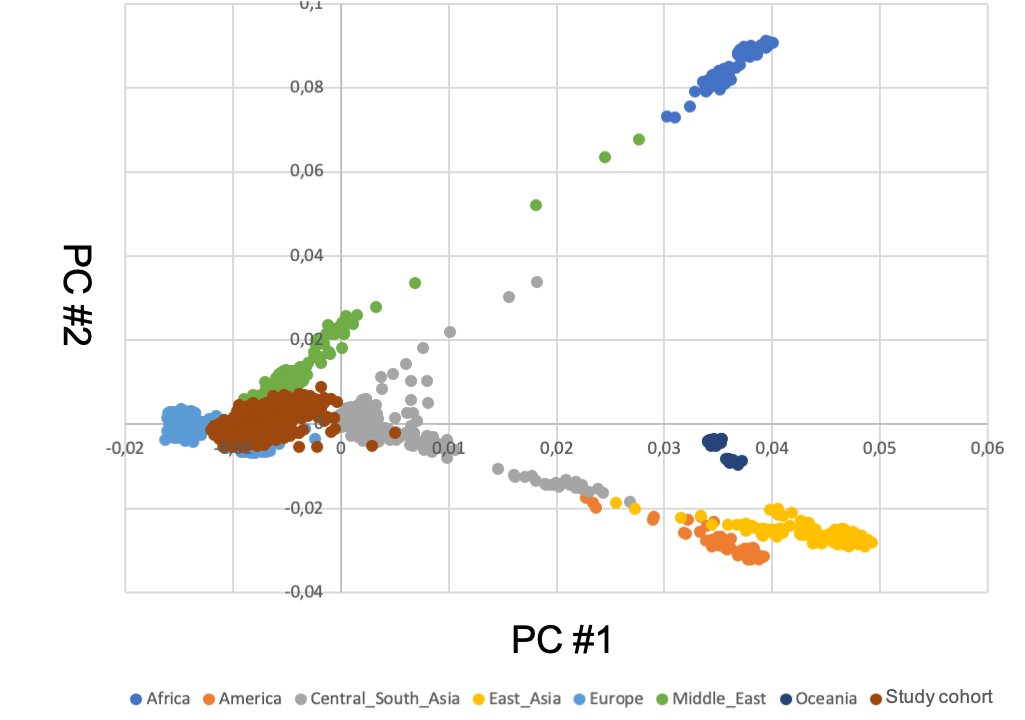


Figure S2: Scatter plot of first 2 principal components of all 3,649 study participants in comparison to various 1KGP populations. Plot confirms the overlap of the European cohort and the study cohort.

### Figure S3: GWAS results for levetiracetam in focal epilepsies, QQ-plot and Manhattan plot


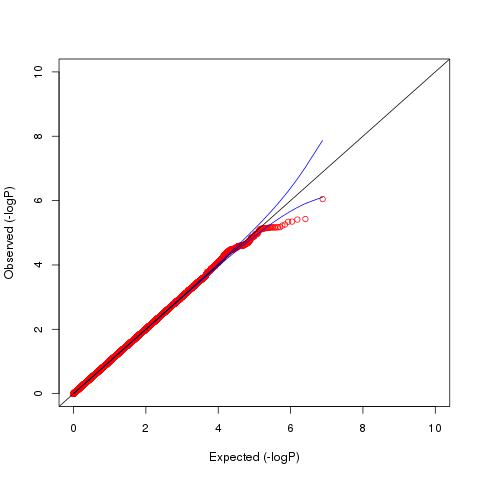


𝛌=0.994


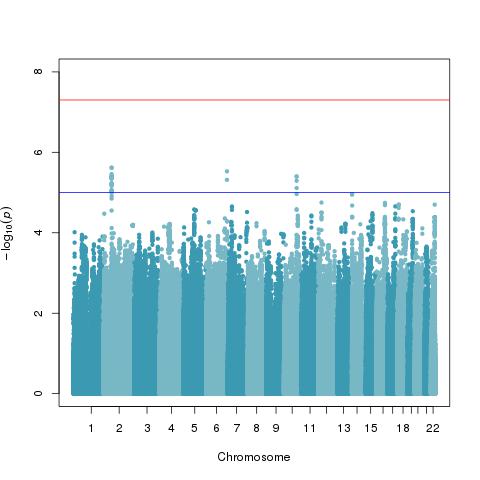


2p15, *TMEM17*

6q27

10q24.2

### Figure S4: GWAS results for lamotrigine in focal epilepsies, QQ-plot and Manhattan plot


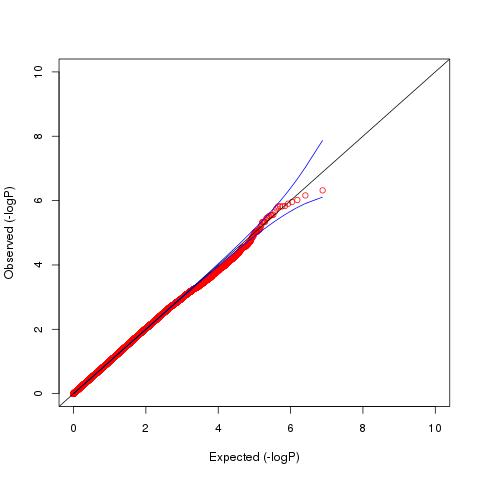


𝛌=1.011


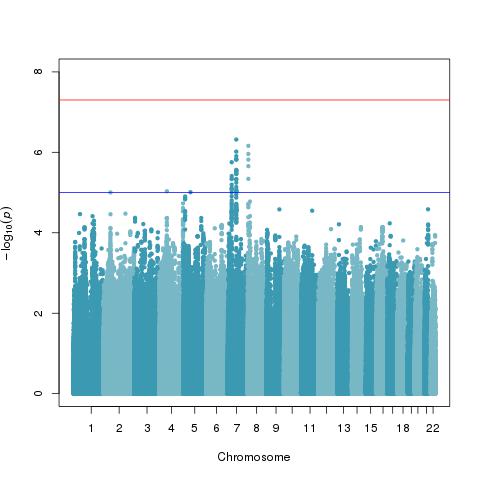


7p14.3, *PDE1C*

7q11.22

8p23.2, CSMD1

### Figure S5: GWAS results for carbamazepine in focal epilepsies, QQ-plot and Manhattan plot


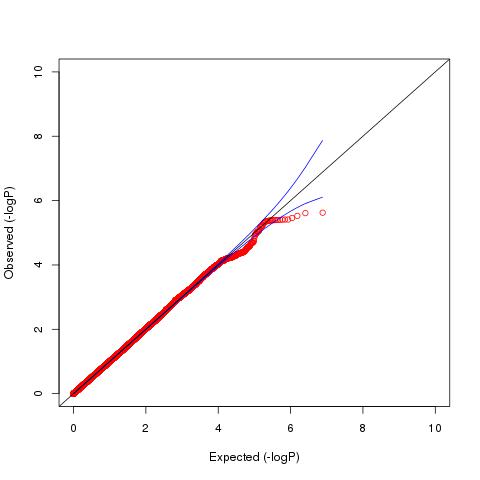


𝛌=1.009


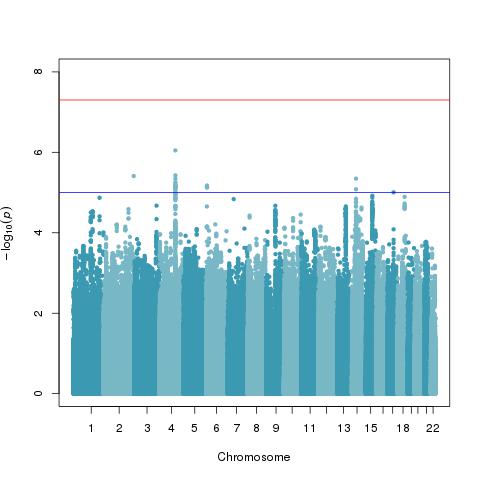


2q37.3

4q28.1

14q22.1, TRIM9

### Figure S6: GWAS results for oxcarbazepine in focal epilepsies, QQ-plot and Manhattan plot


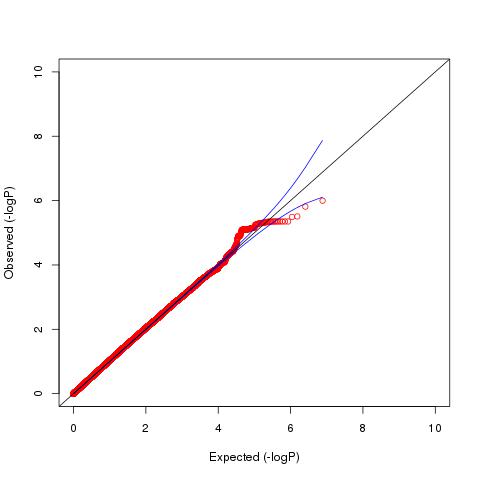


𝛌=1.053


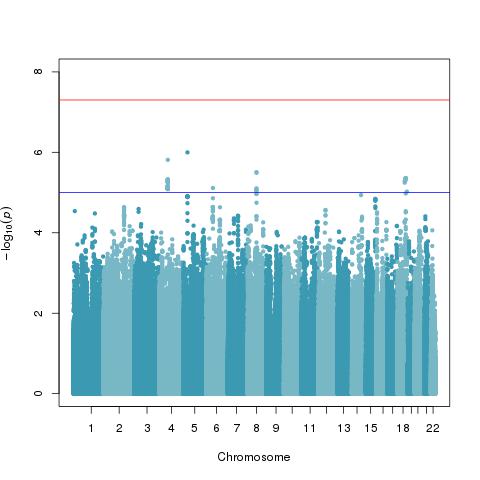


4q13.2

5p13.3

8q13.2, C8orf34

18q22.1

### Figure S7: GWAS results for phenytoin in focal epilepsies, QQ-plot and Manhattan plot


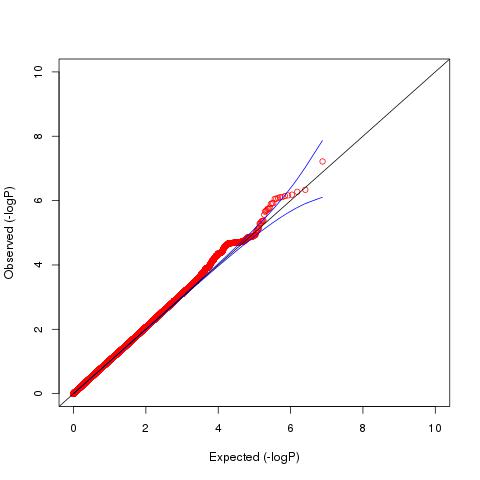


𝛌=1.064


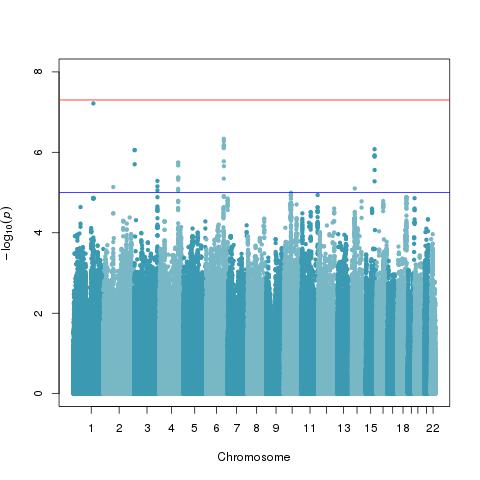


15q26.1, SV2B

6q24.1

3p26.2

1q24.2

### Figure S9: GWAS results for calcium channel-active ASMs in focal epilepsies, QQ-plot and Manhattan plot


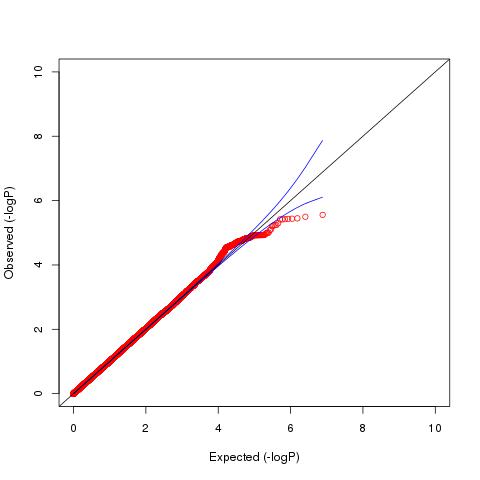


𝛌=1.014


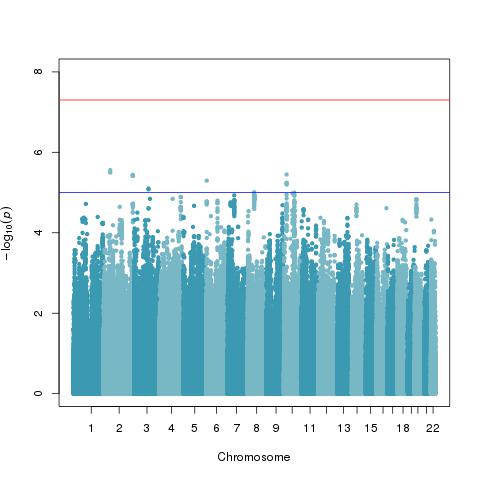


10p12.31

2q37.1, SP140

2p16.3

### Figure S10: GWAS results for valproic acid in focal epilepsies, QQ-plot and Manhattan plot


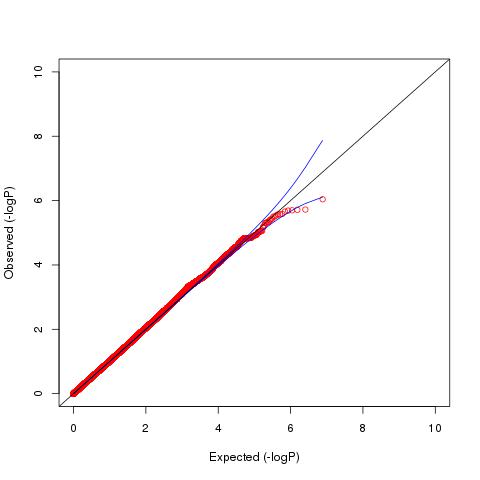


𝛌=1.019


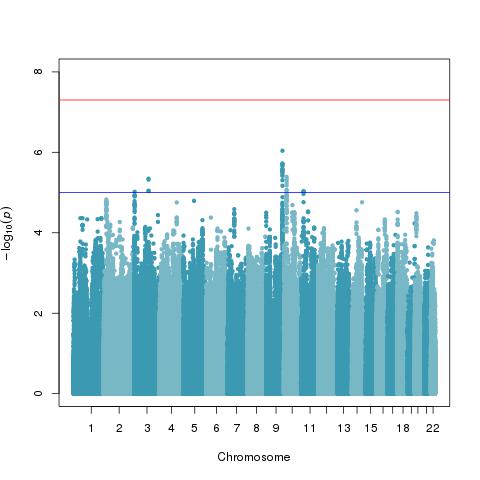


10p12.31

9q33.3, PBX3

3q13.2

### Figure S11: GWAS results for levetiracetam in all epilepsies, QQ-plot and Manhattan plot


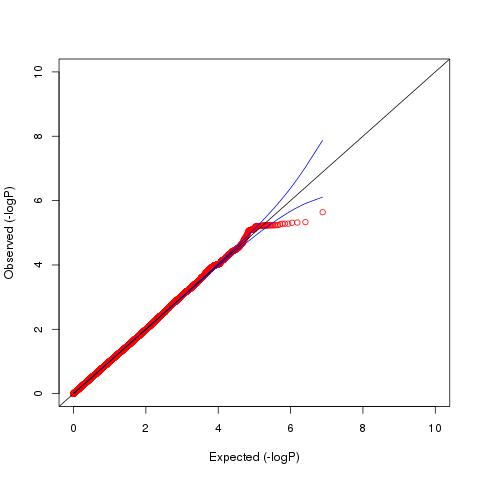


𝛌=1.000

4q28.1


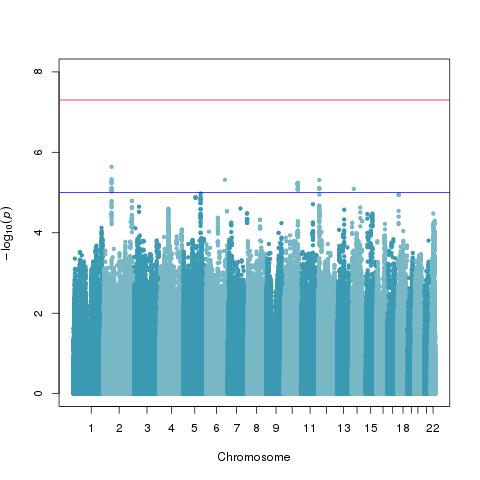


2p15, *TMEM17*

6q24.3

### Figure S12: GWAS results for lamotrigine in all epilepsies, QQ-plot and Manhattan plot


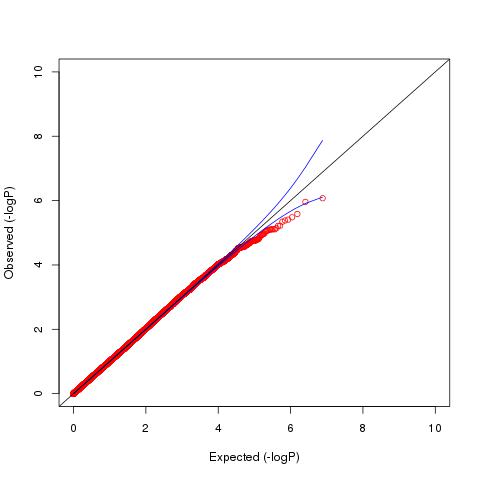


𝛌=1.005


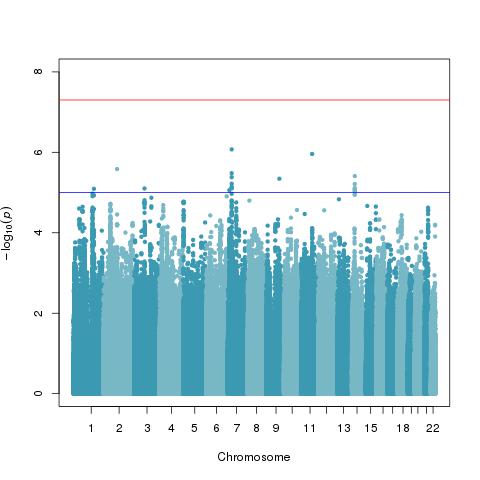


7p14.3, PDE1C

2q12.2

9q31.1, GRIN3A

14q21.1

### Figure S13: GWAS results for calcium channel-active ASMs in all epilepsies, QQ-plot and Manhattan plot


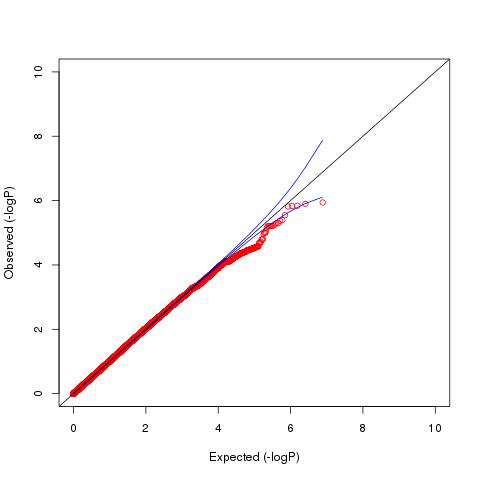


𝛌= 1.006


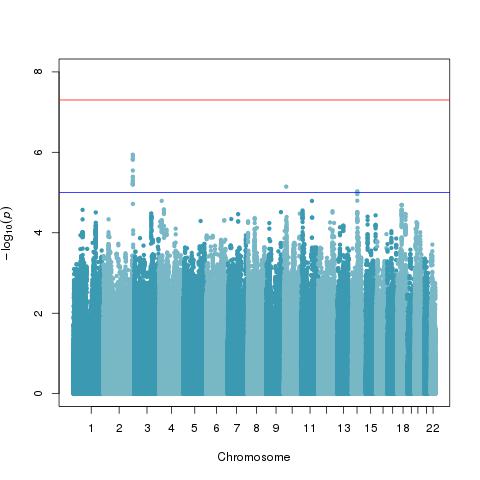


2q37.1, SP140

### Figure S12: GWAS results for valproic acid in all epilepsies, QQ-plot and Manhattan plot


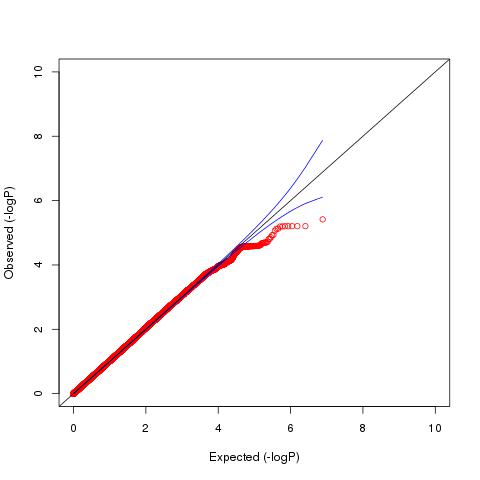


𝛌=1.019


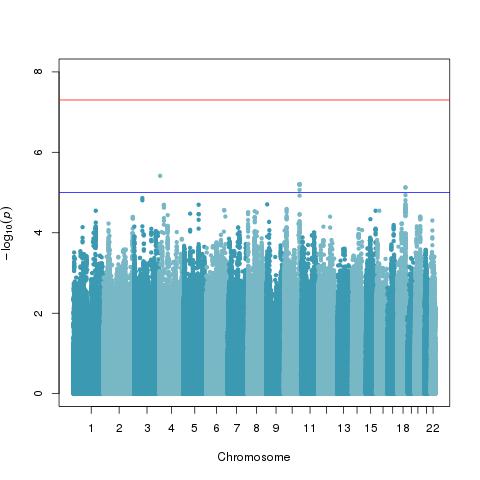


4p16.1
